# Drug-drug interaction between ensitrelvir and tacrolimus in a patient undergoing treatment for COVID-19: a case report

**DOI:** 10.1186/s40780-025-00411-y

**Published:** 2025-01-22

**Authors:** Yuki Miyata, Ryo Yamaguchi, Takehito Yamamoto, Toshiyuki Kishida, Kazuhiko Ikeuchi, Hiroaki Harada, Takeya Tsutsumi, Keishi Fujio, Tappei Takada

**Affiliations:** 1 Department of Pharmacy, The University of Tokyo Hospital, 7-3-1 Hongo, Bunkyo-Ku, Tokyo, 113-8655 Japan; 2 Department of Infectious Diseases, The University of Tokyo Hospital, Tokyo, Japan; 3 Department of Infection Control and Prevention, The University of Tokyo Hospital, Tokyo, Japan; 4 Department of Allergy and Rheumatology, Graduate School of Medicine, The University of Tokyo, Tokyo, Japan

**Keywords:** Ensitrelvir, Tacrolimus, COVID-19, Drug-drug interaction, Cytochrome P450 3A, P-glycoprotein

## Abstract

**Background:**

Ensitrelvir is a novel SARS-CoV-2 3-chymotrypsin-like protease inhibitor, similar to nirmatrelvir/ritonavir. Several case reports have demonstrated the efficacy of 3-chymotrypsin-like protease inhibitors in treating prolonged coronavirus disease 2019 (COVID-19) in immunocompromised patients. Tacrolimus (TAC) is a widely used immunosuppressive agent whose blood level can increase significantly due to the inhibition of cytochrome P450 3A (CYP3A) and P-glycoprotein by nirmatrelvir/ritonavir. Since ensitrelvir also inhibits CYP3A and P-gp, similar elevations in TAC levels are expected. A prior case report observed an increase in TAC trough levels with concurrent administration of ensitrelvir. However, no studies have quantitatively described the changes in TAC blood levels and clearances before and after ensitrelvir administration when TAC administration was discontinued to mitigate the drug-drug interaction (DDI) risk; data on safe dosing protocols to avoid the DDI during co-administration of ensitrelvir and TAC remain lacking. Here, we report a case in which TAC levels were successfully managed in a patient with rheumatoid arthritis (RA) who received ensitrelvir for persistent COVID-19 by preemptive discontinuation of TAC and close monitoring of TAC blood levels following ensitrelvir administration.

**Case presentation:**

An 81-year-old Japanese woman who had been administered TAC (1.5 mg once daily) for RA received two courses of remdesivir for moderate COVID-19. However, her viral load remained high and her respiratory status deteriorated. Considering persistent COVID-19, we initiated combination therapy with remdesivir and ensitrelvir (day 0). TAC was discontinued, and the TAC blood levels decreased from 3.6 ng/mL to 1.1 ng/mL over five days. Subsequently, we re-administered TAC (0.2 mg), observing a level of 1.0 ng/mL by day 7. The TAC dose was adjusted to 1.0 mg daily, and TAC levels on day 12 and 14 were 6.5 and 3.7 ng/mL, respectively. TAC (1.5 mg daily) was resumed on day 15. The calculated t_1/2_ of TAC were 33.7, 71.9, and 114.6 h from day -1 to 0, day 0 to 2, and day 2 to 5, respectively. The t_1/2_ of TAC was extended to 3.4-fold its original duration under ensitrelvir treatment.

**Conclusions:**

This DDI extended the half-life of TAC by approximately 3.4-fold, an effect that gradually diminished over 7 to 10 days. When patients receiving TAC treatment start ensitrelvir therapy, a dose reduction of TAC by approximately one-third to one-fourth is considered appropriate.

**Supplementary Information:**

The online version contains supplementary material available at 10.1186/s40780-025-00411-y.

## Introduction

The coronavirus disease 2019 (COVID-19) pandemic has significantly impacted the global health system. COVID-19 has caused a substantial number of deaths, which continue to rise [1]. Since the beginning of the pandemic, several drugs and vaccines have been developed to combat COVID-19 pandemic. Indeed, antiviral drugs with various mechanisms of action have been developed over the last three years [2].

Ensitrelvir is a 3-chymotrypsin-like protease inhibitor, similar to nirmatrelvir/ritonavir (NMV/RTV), which reduces the viral load of severe acute respiratory syndrome coronavirus 2 (SARS-CoV-2) in a dose-dependent manner [3, 4]. In the post-vaccine era, clinical trials of ensitrelvir have shown a reduction in the duration of mild-to-moderate COVID-19 symptoms caused by the Omicron variant [3]. Post-marketing big data studies also suggest that it may prevent the progression to severe COVID-19 in outpatients [5]. In contrast to NMV/RTV, ensitrelvir is administered orally once daily regardless of mealtime because of its long half-life (approximately 50 h) and can be used in patients with renal impairment [6, 7].

Antivirals are usually administered in the early stages of COVID-19 to prevent disease progression; however, persistent infection with SARS-CoV-2 can occur in some immunocompromised patients [8]. A treatment strategy for persistent COVID-19 has not yet been established. However, several case reports have shown the efficacy of remdesivir combined with NMV/RTV or ensitrelvir in remdesivir-refractory COVID-19 [9].

From the pharmacokinetic aspect, several in vitro and in vivo studies have shown that ensitrelvir strongly inhibits Cytochrome P450 3A (CYP3A), P-glycoprotein (P-gp), and several other transporters including the breast cancer resistance protein (BCRP) [10, 11]. However, because ensitrelvir has been approved by the Special Approval for Emergency system, data from drug-drug interactions (DDI) trials are limited. Therefore, the risks and clinical impacts of DDI with unexplored concomitant drugs warrant further consideration.

Tacrolimus (TAC) is a calcineurin inhibitor used to treat many diseases, such as solid organ transplant recipients, rheumatoid arthritis (RA), and other autoimmune diseases [12]. TAC is a substrate of CYP3A4/5 and P-gp [13], necessitating caution when co-administered with inhibitors of these enzymes and transporters, including anti-COVID-19 drugs. For instance, co-administration of NMV/RTV led to a significant elevation in TAC blood levels, even after discontinuation of NMV/RTV, resulting in acute kidney injury [14, 15]. Since ensitrelvir also inhibits CYP3A and P-gp, TAC levels are expected to elevate. In fact, a previous case report has documented an approximate three-fold increase in TAC trough levels with concurrent ensitrelvir administration [16]. However, no studies have described the temporal profile of TAC blood level changes before and after ensitrelvir administration when TAC was preemptively discontinued to mitigate DDI risk. Consequently, data on safe dosing protocols to avoid DDI during the therapeutically necessary co-administration of ensitrelvir and TAC remain lacking. Here, we report a case in which TAC levels were successfully managed in a patient with RA who received ensitrelvir for persistent COVID-19, by discontinuing TAC prior to ensitrelvir administration; in this case, we estimated the magnitude of DDI to set the timing of TAC discontinuation. Further, we closely monitored TAC blood levels after administration of ensitrelvir to determine the timing and dosage of TAC restart.

## Case presentation

An 81-year-old Japanese woman who had been diagnosed with RA-associated interstitial lung disease was followed up at The University of Tokyo Hospital. The patient had a history of secondary Sjögren's syndrome, dementia, diabetes, and hypertension and was treated with prednisolone (3 mg per os (PO), once daily after breakfast), abatacept (500 mg intravenous injection (IV), once monthly), and oral TAC. TAC was started 4 years ago at a dose of 2 mg daily, achieving a TAC trough level of 6.6 ng/mL after 5 days. The dose was reduced to 1.5 mg daily five months later due to increased serum creatinine (Scr). Subsequently, TAC dose was maintained at 1.5 mg daily without obvious adverse effects, and Scr levels were maintained within normal range (0.75—1.0 mg/dL). On day -30 (30 days before the start of ensitrelvir treatment), the patient complained of fatigue, sore throat, and productive cough at a routine outpatient visit. The patient tested positive for SARS-CoV2 antigen and was admitted emergently with clinically and radiologically confirmed pneumonia.

On admission, the patient had a temperature of 36.4℃, oxygen saturation (SpO2) of 97% on room air, blood pressure of 108/70 mmHg, and heart rate of 89 beats per minute. On the day of admission, the patient was started on a five-day course of remdesivir (200 mg IV once, followed by 100 mg IV every 24 h for 4 days). The patient was also administered corticosteroid (day -30 to -21, dexamethasone 6 mg PO, once daily after breakfast; day -20 to -17, prednisolone 30 mg PO, once daily after breakfast). On day -23, baricitinib (2 mg PO, once daily after breakfast for 6 days) was administered. On day -17, methylprednisolone (1,000 mg IV, once daily for 5 days) was administered, followed by dexamethasone (6 mg IV, once daily after breakfast). The cycle threshold (Ct) value from the SARS-CoV2 PCR after treatment (day 15) was 25.73, indicating persistent viral presence; therefore, a second five-day course of remdesivir was administered. On day -13, tocilizumab (360 mg IV, once daily) was administered. On day -7, the Ct value was 26.18, the viral load remained high, and a computed tomography (CT) scan showed worsening of pneumonia and respiratory status. Consequently, a third course of remdesivir was initiated on day -2 (200 mg IV once, followed by 100 mg IV daily for 9 days). On day 0, the patient was started on ensitrelvir treatment (375 mg PO once, followed by 125 mg PO for 4 days) in combination with remdesivir. At the time of the start of ensitrelvir therapy (day 0), the patient was taking the following medications: TAC (1.5 mg PO, once daily after dinner), dexamethasone (6 mg IV, once daily), sulfamethoxazole/trimethoprim (400 mg/80 mg PO, once daily after breakfast), rosuvastatin (5 mg PO, once daily after breakfast), nicorandil (5 mg PO, 3 times daily after meals), aspirin (100 mg PO, once daily after breakfast), esomeprazole (20 mg PO, once daily after breakfast), rivastigmine patch (18 mg, once daily), and subcutaneous denosumab (60 mg, every six months). The patient had received a COVID-19 vaccine approximately one year ago. The patient had no supplements or over-the-counter drugs. Grapefruit was not offered during the hospital stay. Before the start of ensitrelvir therapy, a clinical pharmacist informed the attending physician regarding the potential DDI between ensitrelvir and TAC or rosuvastatin (BCRP substrate) and recommended avoiding the concomitant use. Consequently, TAC was discontinued from the day before the start of ensitrelvir (day -1), and TAC blood levels were frequently monitored using a Siemens Dimension EXL200 with Flex Cartridge Tacrolimus (ACMIA method, Siemens). Rosuvastatin treatment was discontinued on day 0. Given that ensitrelvir is metabolized by CYP3A [17], dexamethasone, which reportedly induces CYP3A [18], was replaced with methylprednisolone on day 1.

**Fig. 1:**
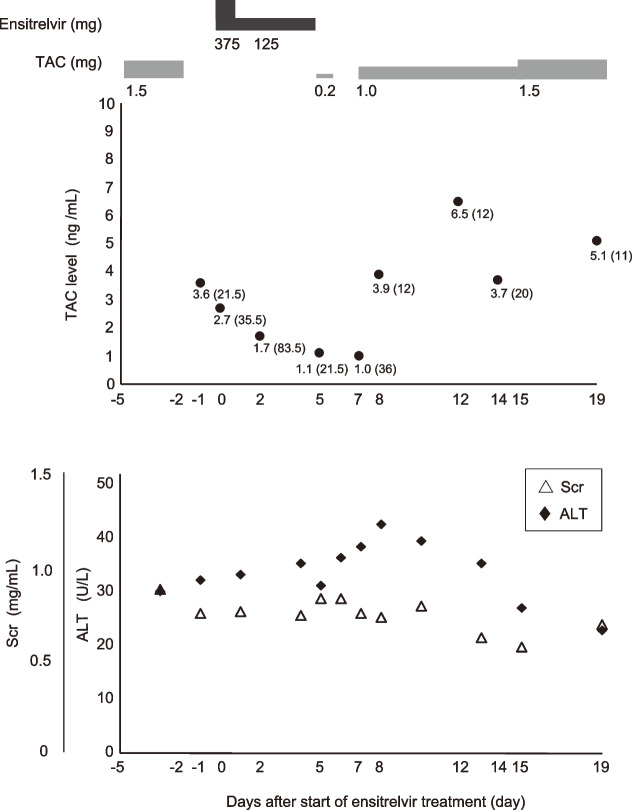
Tacrolimus blood levels in a rheumatoid arthritis patient treated with ensitrelvir. TAC and ensitrelvir doses, and laboratory parameters are presented. TAC, tacrolimus; Scr, serum creatinine; ALT, alanine aminotransferase. The time (h) in parentheses indicates the interval between the last TAC dose and the TDM measurement

On day -1, TAC level at 17:00 (21.5 h after the last dose) was 3.6 ng/mL. On day 0, TAC level was 2.7 ng/mL at 7:00 (35.5 h after the last dose), while on day 2, TAC level was 1.7 ng/mL at 7:00 (83.5 h after the last dose). TAC level on day 5 (the next day after the end of ensitrelvir treatment) was reduced to 1.1 ng/mL at 7:00 (155.5 h after the last dose) (Fig. 1). As TAC level decreased to below the target range, the attending physician decided to restart TAC. Based on previous reports on DDI between NMV/RTV and TAC [14, 15], we considered that ensitrelvir inhibits CYP3A and P-gp not only in the liver but also in intestine and increases the bioavailability (F) of TAC: Therefore, the clinical pharmacist recommended a TAC dose of 0.2 mg PO daily, assuming a volume of distribution (V_d_) of 42 L (1 L/kg, based on a body weight of 42 kg) [19], and F of 100% (assuming complete inhibition of intestinal CYP3A and P-gp), allowing an increase in TAC blood level up to 5 ng/mL Following the suggestion by the clinical pharmacist, 0.2 mg of TAC was administered orally after dinner (19:00) on day 5. On day 7 at 7:00, TAC level was 1.0 ng/mL. Because TAC blood level did not increase as calculated, the inhibitory effects of ensitrelvir on CYP3A and P-gp in the gastrointestinal tract seemed to be minimal. Therefore, 1.0 mg of TAC was administered orally at 19:00 on day 7. On day 8, TAC level at 7:00 was 3.9 ng/mL, which was similar to that before ensitrelvir treatment (day -1); thus, TAC was continued at a dose of 1.0 mg daily until day 14. TAC levels were 6.5 ng/mL at 7:00 on day 12 (12 h after the last dose) and 3.7 ng/mL at 15:00 on day 14 (20 h after the last dose). On day 15, assuming that ensitrelvir was almost completely cleared from the body and that the effects of the DDI had resolved, we reset TAC dose to 1.5 mg daily. During ensitrelvir treatment, alanine aminotransferase (ALT) and Scr levels remained within normal ranges (Fig. 1). The SARS-CoV-2 PCR Ct values on days 0, 5, 12, and 19 were 25.88, 30.03, 34.36, and 34.89, respectively, indicating a decrease in the viral load. On day 18, CT imaging showed improved inflammation in both lung fields. However, oxygen demand showed little improvement.

Assuming a one-compartment model, the elimination rate constant (k_e_), elimination half-life (t_1/2_), and maximum plasma concentration (C_max_) of TAC were calculated as follows:

$${\mathrm k}_{\mathrm e}\;=\ln\;\left({\mathrm C}_1/{\mathrm C}_2\right)\;/\;\left({\mathrm T}_2-{\mathrm T}_1\right)$$

$${\mathrm t}_{1/2}\;=\;0.693/{\mathrm k}_{\mathrm e}$$

$${\mathrm C}_{\max}={\mathrm C}_{\mathrm{trough}}+\mathrm F\ast\mathrm{Dose}\ /\ {\mathrm V}_{\mathrm d}$$

where C_1_ and C_2_ represent the TAC blood levels at times T_1_ and T_2_ in the same dosing interval (T_2_ > T_1_), respectively. C_trough_ represents TAC blood levels immediately before administration and dose represents the TAC dose_._ The volume of distribution (V_d_) was assumed to be 42 L (1 L/kg based on a body weight of 42 kg). If the C_trough_ was unavailable, C_trough_ was estimated using k_e_.

The calculated t_1/2_ of TAC was 33.7, 71.9, and 114.6 h from day -1 to 0, day 0 to 2, and day 2 to 5, respectively.

## Discussion

In this case report, we successfully managed the TAC level in a COVID-19 patient who received ensitrelvir therapy during TAC treatment for RA. Pharmacokinetic analyses utilizing consecutive TAC blood levels revealed that treatment with ensitrelvir led to up to a 3.4-fold prolongation of the t_1/2_ of TAC; The t_1/2_ of TAC before taking ensitrelvir (day-1 to 0) was 33.7 h, which was similar to the reported t_1/2_ of 32 h [20], and this DDI resolved in approximately 7 to 10 days. We believe that our report provides clinically important insights into the dose adjustment of TAC when co-administered with ensitrelvir.

In this patient, t_1/2_ of TAC was prolonged after the start of ensitrelvir treatment, extending from 33.7 h between days -1 and 0 to 71.9 h from days 0 to 2, and reaching 114.6 h from days 2 to 5. The t_1/2_ of TAC was extended to 3.4-fold its original duration under ensitrelvir treatment. Shimizu et al. reported that ensitrelvir increased the area under the blood concentration–time curve (AUC) of midazolam (a typical CYP3A4 substrate) by 6.7-fold [11]. Based on this report, the predicted AUC increase in TAC with ensitrelvir co-administration was calculated to be 2.59-fold using the CR-IR method described by Ohno et al. [21, 22]. This estimate aligns with the observed prolongation of the t_1/2_ of TAC. In this case, the impact of TAC on the absorption process was considered negligible because TAC was withdrawn before initiating the ensitrelvir treatment. Therefore, the inhibition of CYP3A in the liver by ensitrelvir likely caused a threefold increase in the AUC of TAC. Recently, Naganawa et al. reported that the co-administration of ensitrelvir increased TAC blood level by approximately threefold [16]. This observation is consistent with the threefold increase in the t_1/2_ of TAC observed in this case report. Interestingly, the magnitude of the DDI between ensitrelvir and TAC (approximately threefold) was more moderate than that between NMV/RTV and TAC. Although further clinical studies are needed, ensitrelvir would be a good alternative to NMV/RTV in patients treated with CYP3A substrate drugs pharmacologically.

During the discontinuation of TAC, its blood levels gradually decreased. By 7:00 on day 5, TAC level had fallen to 1.1 ng/mL, approximately one-third of the level before the start of ensitrelvir treatment, leading us to consider restarting TAC administration. Because there were no in vivo DDI data between ensitrelvir and TAC, we calculated a TAC dose of 0.2 mg daily, assuming that intestinal CYP3A and P-gp were completely inhibited by ensitrelvir, to mitigatethe risk of adverse effects due to increased TAC exposure. However, after administering 0.2 mg TAC at 19:00 on day 5, TAC level at 7:00 on day 7 was only 1.0 ng/mL (36 h post-administration), which was lower than the expected level (5 ng/mL). A previous DDI study reported that ensitrelvir increased the t_1/2_ and AUC of midazolam by 2.3-fold and 6.7-fold, respectively [11]. Additionally, ensitrelvir reportedly increased the AUC of digoxin, a P-gp substrate [10]. These reports indicated that ensitrelvir significantly inhibits CYP3A and P-gp in both liver and gastrointestinal tract. Contrary to expectations, the effect of ensitrelvir on the intestinal absorption of TAC seems to be limited. Although the underlying mechanism remains unclear, there are two possible reasons for the limited effect of ensitrelvir on the absorption process of TAC. First, the inhibitory effect of ensitrelvir on CYP3A in the gastrointestinal tract was weaker than that of NMV/RTV. Cox et al. reported that NMV/RTV increased the AUC of midazolam by 14.3-fold, which was greater than that caused by ensitrelvir (6.7-fold), although the effects of NMV/RTV and ensitrelvir on the t_1/2_ of midazolam were similar (2.1- and 2.3-fold, respectively) [23]. As midazolam is a pure CYP3A substrate and is not transported by transporters [24], the magnitude of change in the midazolam AUC reflects the extent of CYP3A inhibition. Considering the degree of prolongation in t_1/2_ is similar, the observed difference in the magnitude of increase in midazolam AUC between concomitant use of NMV/RTV and ensitrelvir (14.3- and 6.7-fold, respectively) is likely attributable to the difference in the inhibitory potential for intestinal CYP3A between NMV/RTV and ensitrelvir: the inhibitory potential of ensitrelvir would be less potent than that of NMV/RTV. Second, P-gp inhibition was not observed in the gastrointestinal tract. Studies have shown that when P-gp substrates and inhibitors were used together, the rate of increase in the AUC of the P-gp substrates was lower for groups that took them two hours apart than for those that took them simultaneously [25, 26]. In the present case, there was a 33.5 h gap between the last dose of ensitrelvir and the resumption of TAC, suggesting that the effect of P-gp inhibition by ensitrelvir in the gastrointestinal tract was minimal. It should be noted that the degree of DDI observed in this case may not apply to other cases. Further investigations are needed to determine the effect of ensitrelvir on the absorption process under conditions that may strongly inhibit P-gp (e.g., co-administration with TAC).

Next, we evaluated the duration of DDI using ensitrelvir. The t_1/2_ of ensitrelvir was 51.4 h, suggesting that 7–8 days are required to clear ensitrelvir from the body. Considering that ensitrelvir inhibits CYP3A in a time-dependent and irreversible manner [6, 11], the inhibitory effects of ensitrelvir may persist for several days even after ensitrelvir is cleared from the body. Assuming that the t_1/2_ of TAC from days 2–5 (114.6 h) continued on days 5–8, we simulated the TAC level trends using a one-compartment model with various F values (1, 0.5, 0.25, 0.125, and 0.05) to obtain a reasonable estimate of F after ensitrelvir administration (Supplemental Table S1). Consequently, when F was set to 0.125, the simulated TAC levels matched the measured levels at 7:00 on day 8 (Supplemental Table S1). The estimated F value of TAC (0.125) was similar to that reported in a previous report (0.25 ± 0.20) [27], which indicates the limited effect of ensitrelvir on the intestinal absorption of TAC. The estimated t_1/2_ for the intervals from day 8 to 12 and from day 12 to 14 were 39.9 h and 23.4 h, respectively (Supplemental Table S1). The simulation results suggested that the impact of DDI by ensitrelvir gradually diminished from days 8 to 12 (4 to 8 days after ensitrelvir discontinuation), although it did not return to the levels observed before the administration of ensitrelvir. In contrast, the t_1/2_ during days 12–14 (23.4 h) returned to levels similar to those before the start of ensitrelvir (33.7 h). These pharmacokinetic analyses indicate that the DDI effect of ensitrelvir gradually diminishes over 7–10 days after its discontinuation. Considering that ensitrelvir irreversibly inhibits CYP3A and has a long t_1/2_ (51.4 h), it is reasonable that the inhibitory effect of ensitrelvir on CYP3A persisted for approximately 10 days. Therefore, caution regarding DDI with ensitrelvir should be exercised for 7–10 days after discontinuation of ensitrelvir. Furthermore, in the case of irreversible CYP3A inhibition, the timing of CYP3A expression recovery varies among individuals [28]. Therefore, TAC after ensitrelvir treatment should be restarted at a reduced dosage and its blood levels should be closely monitored.

The limitations of this study are as follows. First, the F value of the TAC was estimated by simulation without actual C_max_ values. Second, the possible influence of factors other than DDI on the TAC blood levels was not evaluated. TAC levels fluctuate because of various factors, including changes in hepatic function, Hct levels, food intake, the presence of inflammatory pathologies and CYP3A5 genetic polymorphisms [29–31]. Although we confirmed no significant changes in liver enzymes, Hct levels, CRP levels, and dietary intake in this case, we cannot rule out the possibility that the factors not assessed in this case influenced TAC blood levels.

In conclusion, the concomitant use of ensitrelvir decreased the hepatic clearance of TAC and increased the t_1/2_ of TAC by 3.4-fold. The inhibitory effects of ensitrelvir gradually diminished over 7–10 days after discontinuation of ensitrelvir. When administering ensitrelvir to patients treated with TAC, a dose reduction of approximately one-third to one-fourth is recommended, and TAC blood levels should be closely monitored for at least 7–10 days to titrate the dose of TAC.

## Supplementary Information

Supplementary Material 1: Supplemental Table S1. Observed and estimated tacrolimus blood levels after the 5 days of ensitrelvir treatment.

## Data Availability

No datasets were generated or analysed during the current study.

## Abbreviations

COVID-19: Coronavirus disease 2019
TAC: Tacrolimus
NMV/RTV: Nirmatrelvir/ritonavir
CYP3A: Cytochrome P450 3A
P-gp: P-glycoprotein
BCRP: Breast cancer resistance protein
DDI: Drug-drug interactions
SARS-CoV2: Severe acute respiratory syndrome coronavirus 2
CT: Computed tomography
RA: Rheumatoid arthritis
AUC: Area under the curve
Scr: Serum creatinine
SpO2: Oxygen saturation
Ct: Cycle threshold
PO: Per os
IV: Intravenous injection
