# Supplementary material for: Drug-drug interaction between ensitrelvir and tacrolimus in a patient undergoing treatment for COVID-19: a case report
Source: J Pharm Health Care Sci. 2025 Jan 22;11:3. doi: 10.1186/s40780-025-00411-y (PMC11756168; doi:10.1186/s40780-025-00411-y)
Supplement: Supplementary file 1 — Supplementary Material 1: Supplemental Table S1. Observed and estimated tacrolimus blood levels after the 5 days of ensitrelvir treatment. [file 40780_2025_411_MOESM1_ESM.docx]

**Supplemental Materials**

Table S1: Observed and estimated tacrolimus blood level after the 5 days of ensitrelvir treatment.

| Time course | TAC  Dose (mg) | Estimated  TAC level (ng/mL)^a^ | | | | | Observed  TAC level  (ng/mL) | Estimated TAC half-life (hr) |
| --- | --- | --- | --- | --- | --- | --- | --- | --- |
|  |  | Bioavailability | | | | |  |  |
|  |  | 1 | 0.5 | 0.25 | **0.125** | 0.05 |  |  |
| Day5, 7:00 |  |  |  |  |  |  | 1.1 | 114.6^b^ |
| Day5, 19:00 (trough) |  | 1.0 | 1.0 | 1.0 | **1.0** | 1.0 |  |  |
| Day5, 19:00 (peak) | 0.2 | 5.8 | 3.4 | 2.2 | **1.6** | 1.3 |  |  |
| Day7, 7:00 |  | 4.7 | 2.7 | 1.8 | **1.3** | 1.0 | 1.0 |  |
| Day7, 19:00 (trough) |  | 4.3 | 2.5 | 1.7 | **1.2** | 0.9 |  |  |
| Day7, 19:00 (peak) | 1 | 28.1 | 14.5 | 7.6 | **4.2** | 2.1 |  |  |
| Day8, 7:00 |  | 26.2 | 13.4 | 7.1 | **3.9** | 2.0 | 3.9 |  |
| Day8, 19:00 (trough) | 1 | - | - | - | **3.2** | - |  | 39.9^c^ |
| Day8, 19:00 (peak) |  | - | - | - | **6.1** | - |  |  |
| Day9, 19:00 (trough) | 1 | - | - | - | **4.0** | - |  |  |
| Day9, 19:00 (peak) |  | - | - | - | **7.0** | - |  |  |
| Day10, 19:00 (trough) | 1 | - | - | - | **4.6** | - |  |  |
| Day10, 19:00 (peak) |  | - | - | - | **7.6** | - |  |  |
| Day11, 19:00 (trough) | 1 | - | - | - | **5.0** | - |  |  |
| Day11, 19:00 (peak) |  | - | - | - | **8.0** | - |  |  |
| Day12, 7:00 |  | - | - | - | **6.5** | - | 6.5 |  |
| Day12, 19:00 (trough) | 1 | - | - | - | **4.5** | - |  | 23.4^c^ |
| Day12, 19:00 (peak) |  | - | - | - | **7.5** | - |  |  |
| Day13, 19:00 (trough) | 1 | - | - | - | **3.7** | - |  |  |
| Day13, 19:00 (peak) |  | - | - | - | **6.7** | - |  |  |
| Day14, 15:00 |  | - | - | - | **3.7** | - | 3.7 |  |

^a^ The tacrolimus level was estimated using a one-compartment pharmacokinetics model for oral administration, without considering the absorption rate constant. For tacrolimus levels after day 8, only values predicted with F = 0.125 are shown.

^b^ We hypothesize that the half-life of tacrolimus, which is 114.6 hr just before the discontinuation of ensitrelvir, continues until day 8.

^c^ Assuming a bioavailability of 0.125, we estimated the half-life for each interval based on the TAC observed values.

Abbreviation; TAC, Tacrolimus
